# Report of Representatives from Rock Island Medical Society on the Proceedings of the State Medical Society

**Published:** 1857-09

**Authors:** E. K. Bowman


*Report of Representatives from Rock Island Medical Society on the Proceedings of the State Medical Society.* By E. K. Bowman.

Your representatives, elected to represent this Society in the State Medical Society, which held its sessions at Chicago on the 2d, 3d and 4th days of June, have to report that, at the time appointed, they repaired to the place indicated, and took such part in the proceedings as seemed necessary. Finding that Rock Island had never been represented in the State organization, we were not slow to perceive that we were rather regarded as from the unenlightened districts, where medical science had but a slender number of votaries. However, we were duly encouraged by the expressions of pleasure, at the evidence afforded by our presence, that some interest was awakened in our region. The sum of our conclusions did not entirely overwhelm self-esteem, nor force on us the overshadowing conviction that the State Society combined *all* the wisdom and science of the State. We thought it valuable, however, as a bond of union for the profession and the medical organizations of the counties. The discussions on various papers, if not profound, were suggestive and useful, giving the various kinds of treatment in different hands and localities. A paper read by Dr. Bailey, of Joliet, on Congestive Intermittents elicited considerable discussion, but your representatives were not able, giving vigilant attention, to discover any new truth, either in cause, pathology or treatment, developed by the paper or discussion. A paper was read by Dr. Goodbrake, of Clinton, on the medical properties of Asclepias Tuberosa, giving an account of several cases in which, as he supposed, it was successful in bowel complaints, but always (if I observed correctly) while in the administration of other and appropriate remedies. It seemed to your representatives, from the great volume of decoction of the root used, that its main value consisted in determining to the skin, and that any warm diluent or demulcent that does not offend the stomach will probably accomplish as much. We were not converted to such strong faith in Asclepias Tuberosa, that, if the life of a child were in danger, we would feel safe in relying on it as a sufficient resource. Indeed, in encountering this formidable scourge in July, August and September, we need to be armed with all the accuracy of judgment which science, skill and experience can give, and have at our command all the most reliable agents with which nature and art have furnished us.

Dr. Brainard addressed the Society on the subject of ununited fractures, giving his views of treatment and the most efficient means of effecting a cure. You are probably aware that his treatment consists in perforating through the soft parts with an instrument somewhat resembling a trochar, and wounding the broken surfaces so as to excite the secretion of bone, withdrawing the instrument so as not to admit the air, and avoiding such damage to soft parts, if possible, as to obviate any danger of suppuration. The plan adopted by Dr. B. probably will be more successful than any heretofore put in practice.

A paper was presented, (ostensibly by the Esculapian Society, located in the south-eastern part of the State,) and read by the author, on the subject of Stomatitis Materni. The most prominent idea which struck your representative, was, that the disease was becoming alarmingly prevalent in our country. The only cause assigned for the assumed fact was the commingling of races, which is going on so extensively on our continent, from foreign emigration. No reasons, physiological, climatic or otherwise was given why amalgamation of races should develope such a distressing affection as Stomatitis Materni. The treatment, as given by the author of the paper, consisted almost wholly in giving, after each meal, large portions of sub. nitrate of bismuth and carb. soda, in combination.

The discussion on this paper was quite interesting. Prof. Blaney made quite an address, giving his views, which were that the disease was caused by the loss of albumen from the maternal system to furnish material for the growth of the child. His remedy, to feed patients on such diet as would furnish the most albumen, to wit: eggs, milk, etc.

Professor Davis, who, by the way, impressed your representatives as a man of thorough research, gave his views at some length, and detailed some cases which had fallen under his care. He had not been able to find specifics, nor control his cases with the ease and promptness which was desirable, and would be accomplished if specifics were attainable.

Your representative thinks, on retrospection, that the influence of malaria, acting on delicate and sensitive mucous and nervous organizations, in combination with pregnancy and lactation, was overlooked, and that no treatment will be so efficacious where this element is overlooked as where its agency is attentively considered. This reflection is caused by a recollection of two cases occurring in my own practice, one of which was treated by local applications mainly, and only with temporary relief; the other, with the idea mentioned above, was treated constitutionally with entire success and without any local treatment whatever.

The subject of croup was also up for discussion, yet with all the anxiety to attain complete control, all who spoke confessed that they knew no infalliable treatment, except one man. His advice was to put croup into the category of subjects exclusively under the control of the old women and apply simply water to the throat, then the profession would not see so many fatal cases. The verdancy and self-complacency precluded any suspicion of the honesty of the recommendation. Prof. Davis recommended the yellow sulphate of mercury (turpeth mineral,) as the most reliable emetic known in croup, combining the advantages of emetic and mercurial.

An invitation having been extended to the society to visit Merey Hospital, which is under the care of the Sisters of Charity, we repaired to the hospital at the hour appointed, and made the tour of the wards accompanied by Prof. Davis whose remarks on the pathology and treatment of the various cases, confirmed our previous impressions concerning him, and contributed much to the interest and pleasure of the visit. Several very interesting surgical cases under the care of Prof. Johnson, were also brought to our notice. A partial report on drugs and medicines was made by Prof. Johnson bringing to our notice the discoveries made within the year in that department. The most important and practical that we recollect were the solvent properties of glycerine and its value as a vehicle for the solution and administration of the vegetable alkaloids. On the whole, one noticeable feature presented itself to the mind of your reporter. It was this, that two very separate and distinct classes of men were represented in the society from the ranks of the profession. One of these seem, to the observer, to be active in moving the machinery, ready with remarks on all occasions, in season or out of season, whether having anything to say or not, more anxious to "figure in the transactions," than possessed of ability to make a creditable figure there. More desirous of notoriety than possessed of patience and toil, to achieve it by delving in the mine of truth, and illuminating themselves and others by its brilliant gems. Much to be compared are they, to the venerable hen, that with large ambition, endeavored to spread herself over thirty-two eggs. No large product of vitality was the result, but on the contrary, considerable embryo vitality extinguished. On the other hand, we found men whose ring gave back the sound of the true metal, men, who, with an exalted conception of their glorious, yet much abused profession, are manfully endeavoring to advance and elevate it. Men of toil and research, patient and observing, who take more delight in the evolution of a new truth from the great storehouse of nature, or the practical application of a truth already discovered, than in all the figurings which could be made in all the volumes of all the societies.

Rockford was chosen as the next place of meeting of the society. Your reporter tried somewhat to get Rock Island as the place, and while making out as we thought a good case, yet the majority thought the claims of Rockford stronger, and so decided.

